# Gut Microbiota: Potential Therapeutic Target for Sickle Cell Disease Pain and Complications

**DOI:** 10.1155/2024/5431000

**Published:** 2024-03-19

**Authors:** Tarimoboere Agbalalah, Doofan Bur, Ezinne JaneFrances Nwonu, Adekunle Babajide Rowaiye

**Affiliations:** ^1^Department of Anatomy, Faculty of Basic Medical Sciences, Baze University, Abuja, Nigeria; ^2^Department of Medical Biotechnology, National Biotechnology Development Agency, Abuja, Nigeria; ^3^Department of Agricultural Biotechnology, National Biotechnology Development Agency, Abuja, Nigeria

## Abstract

**Aim:**

Sickle cell disease has witnessed a 41.4% surge from 2000 to 2021, significantly affecting morbidity and mortality rates, particularly in children from regions with elevated under-5 mortality rates. Gut microbiota dysbiosis is increasingly recognised in SCD, exacerbating complications, particularly chronic pain, marked by significant alterations of proinflammatory bacteria abundance. This review explores the therapeutic potential of *Akkermansia muciniphila* and *Roseburia* spp. in alleviating SCD-related complications, emphasising their roles in maintaining gut barrier integrity, reducing inflammation, and modulating immune responses.

**Method:**

A literature search up to November 2023 using PubMed, MEDLINE, and Google Scholar databases explored SCD pathophysiology, gut microbiota composition, *Akkermansia muciniphila and Roseburia* spp. abundance, pain and gut dysbiosis in SCD, and butyrate therapy.

**Result:**

*A. muciniphila and Roseburia* spp. supplementation shows promise in alleviating chronic pain by addressing gut dysbiosis, offering new avenues for sustainable SCD management. This approach holds the potential for reducing reliance on reactive treatments and improving overall quality of life. This research underscores the pivotal role of the gut microbiome in SCD, advocating for personalised treatment approaches.

**Conclusion:**

Further exploration and clinical trials are needed to harness the full potential of these gut bacteria for individuals affected by this challenging condition.

## 1. Introduction

Sickle cell disease (SCD) is a hereditary haemoglobinopathy consisting of at least one haemoglobin (Hb) S allele expressed as homozygous (HbS/S, most common and severe) and heterozygous (HbS/C, less severe), two phenotypes of sickle beta (*β*) thalassemia (HbS/*β*+ thalassemia and HbS/*β*o_thalassemia), and other rare forms such as HbS/D, HbS/O, and HbS/E [1, 2]. This genetic condition results from a missense variant (rs334) in the Hb subunit *β*-globin (HB*β*) gene, leading to the abnormal polymerisation of red blood cells (RBCs) [3]. The distinctive sickle-shaped RBCs formed during polymerisation cause vaso-occlusion, blocking small blood vessels and triggering recurrent episodes of pain, oxygen deprivation, and multiorgan damage [4]. Vaso-occlusive crises (VOCs) and chronic pain are the primary reasons for hospitalisation among SCD patients, imposing a significant healthcare burden, compromising their quality of life, and increasing morbidity and mortality [5].

The disease affects millions globally, with a 41.4% increase in the global SCD population from 5.46 million in 2000 to 7.74 million in 2021 [6]. It is highly prevalent in sub-Saharan Africa, the Caribbean, the Middle East, India, and Mediterranean countries such as Turkey, Greece, and Italy [6]. SCD prevalence is steadily increasing in Europe, the USA, and the UK due to migration [7, 8]. A significant SCD mortality burden in 2021, reaching nearly 11 times the cause-specific all-age deaths globally, with an estimated 376,000 deaths, particularly impacting children in nations with elevated under-5 mortality rates has been observed [9]. Urgent action is required to address the escalating health crisis of SCD, as the lack of comprehensive strategies poses a significant challenge to achieving Sustainable Development Goals 3.1, 3.2, and 3.4. Recognised as a global public health concern by the World Health Organization, the widespread prevalence of SCD underscores the need for immediate and concerted efforts [10].

The United States Food and Drug Administration recently approved two gene therapies, Casgevy and Lyfgenia, for SCD patients aged 12 and older, with Casgevy utilizing CRISPR/Cas9 technology [11]. However, the $2.2 million cost per person raises accessibility concerns, especially for those in resource-poor regions such as sub-Saharan Africa with a high SCD prevalence. With gene therapies' high costs limiting access, there is a crucial need for cost-effective alternatives. Gut microbiota modulation therapy emerges as a promising and economically feasible option to address SCD complications and chronic pain. In resource-poor areas heavily impacted by SCD, prioritizing cost-effective interventions such as gut microbiota modulation becomes imperative. This approach can potentially improve the well-being of affected individuals without imposing significant financial burdens, thus promoting a more inclusive and sustainable approach to managing SCD.

Growing evidence underscores the significant impact of the gut microbiota in SCD [12], with notable changes in intestinal physiology and microbiome composition [13]. The gut microbiota, vital for metabolism and immunity, is disrupted in SCD patients, leading to dysbiosis, particularly involving proinflammatory bacteria [13]. SCD pathophysiological processes impact bacterial colonisation in the gastrointestinal tract (GIT), exacerbating dysbiosis. This altered gut microbiota potentially worsens SCD pathology. Studies suggest a significant role of the gut microbiota and associated metabolites in chronic pain and SCD-related complications [14]. Identifying specific bacterial species with the potential to alleviate chronic pain and manage SCD issues is crucial. Thus, *Akkermansia muciniphila* (*A. muciniphila*) and *Roseburia* spp. could be promising candidates for SCD therapy.

*A. muciniphila* contributes to gut health by preserving barrier integrity, enhancing mucin production for thicker mucus, influencing tight junctions, and reducing inflammation—offering relief from chronic pain [14–17]. *Roseburia* spp., a key butyrate-producing bacterial group, produces the anti-inflammatory short-chain fatty acid (SCFA) butyrate [18]. This compound induces fetal haemoglobin (HbF) production [19], holds immunomodulatory potential [20], and plays a crucial role in maintaining gut barrier integrity [21]. Given their diminished abundance in SCD, *A. muciniphila* and *Roseburia* spp. emerge as promising targets to potentially alleviate the severity and frequency of SCD-related complications. Thus, this review explores the therapeutic benefits of *A. muciniphila* and *Roseburia* spp. for SCD. We examined existing literature on bacteria, investigating pain relief, barrier strength, and SCD complications. The insights gained could lead to new and personalised approaches for managing this complex disease.

## 2. Literature Search

A comprehensive search of PubMed, MEDLINE, and Google Scholar databases was conducted between September and November 2023 to gather relevant articles exploring the role of gut microbiota in managing chronic pain and SCD-related complications. A collection of words and phrases, including but not restricted to “SCD pathophysiology and gut microbiota,” “Gut microbial composition in SCD,” “Implications of dysbiosis in SCD,” and “Pain and gut dysbiosis in SCD.” In addition, our search focused on “*Akkermansia muciniphila*,” “*Roseburia* spp abundance in SCD,” and “butyrate therapy in SCD.” The search included both original research and review articles, involving both human and animal models. No restrictions were imposed on publication dates, and only articles written in English were considered for inclusion in the search results.

### 2.1. Gut Microbiota and Health

The gut microbiota is a diverse community of bacteria, viruses, fungi, protozoa, archaea, and other single-celled organisms living symbiotically in the GIT [22, 23]. The GIT hosts a vast bacterial population, numbering between 9 and 10 [13, 14]. In the colon alone, a diverse community of 160–500 bacterial species with varied characteristics thrives [24]. Six bacteria phyla including *Firmicutes, Bacteroidetes, Proteobacteria, Actinobacteria, Fusobacteria,* and *Verrucomicrobiota* dominate the gut of healthy adults [25, 26]. Alterations in the microbial composition could lead to a reduction in diversity, which, in turn, may promote the growth of pathogenic bacteria [27]. The gut microbiota maintains host health by regulating nutrient absorption and reinforcement of gut integrity and inhibiting the proliferation of pathogens, while also influencing oxidative stress, metabolism, cognition, and the immune system [28–30].

The gut microbiota communicates with the host through the production of SCFAs such as propionate, butyrate, and acetate. These SCFAs are derived from the breakdown of dietary fibre, in addition to vitamins and immunomodulatory peptides [31]. Notably, SCFAs play a crucial role in maintaining microbial homeostasis by promoting the synthesis of mucin, antimicrobial peptides, and tight junction proteins. They also contribute to the reduction of colonic inflammation and oxidative stress [32, 33]. Furthermore, the composition and diversity of the gut microbiota are subject to various influences, including age, sex, diet, antibiotic use, stress, intestinal function, immune responses, genetic mutations, environmental factors, and diseases [28]. These factors collectively shape the intricate balance of the gut microbiota and its impact on host health.

### 2.2. Gut Microbiota Dysbiosis in SCD

Gut microbiota dysbiosis disrupts the integrity of tight junctions between intestinal cells, leading to a cascade of inflammatory responses, cellular adhesion, and tissue damage. This dysregulation is implicated in the occurrence of VOCs in individuals with SCD [12, 34]. In addition, dysbiosis is linked with a reduction in the production of SCFAs, which are important molecules for gut health [35]. Dysbiosis in SCD is influenced by various factors, including host-specific elements and environmental influences such as diet, xenobiotics (including antibiotics and other drugs), and hygiene practices. It is noteworthy that dysbiosis is associated with a spectrum of health issues, ranging from diabetes, allergies, fatty liver disease, and obesity to inflammatory bowel disease [36, 37]. This emphasises the broad-reaching consequences of an imbalanced gut microbiota on human health.

Gut microbiota dysbiosis has been observed in individuals affected by SCD, including children and adults, particularly involving bacteria known for their strong proinflammatory properties [13, 38, 39]. The imbalance in gut microbes has also been replicated in mouse models of SCD [13, 14, 35, 40], further emphasising the association between gut microbiota changes and the pathophysiology of SCD. Understanding and addressing dysbiosis in SCD not only have implications for VOCs but also for the broader spectrum of health issues associated with an imbalanced gut microbiota.

### 2.3. SCD Pathophysiology and Gut Microbiota Dysbiosis

**Figure 1:**
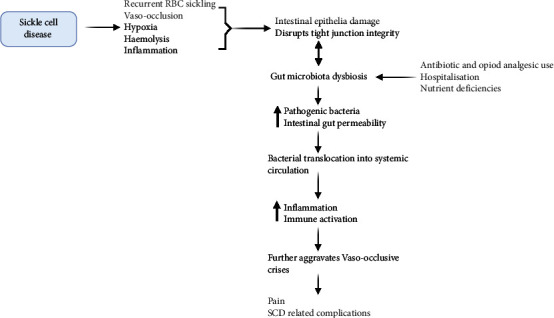
Interaction between SCD pathophysiology and the gut microbiota.

The intricate relationship between SCD pathophysiology and gut dysbiosis involves a complex interplay with significant consequences [13]. SCD is characterised by recurrent sickling RBCs, vaso-occlusion, and hypoxia, affecting the GIT by altering the local environment and influencing bacterial colonisation. This, in turn, leads to damage to the intestinal epithelium and increased gut permeability, thereby weakening the gut barrier and allowing luminal content and bacteria to enter the systemic circulation [13]. Various factors contribute to gut dysbiosis in individuals with SCD, including prolonged antibiotic use, common nutrient deficiencies, and exposure to hospital-associated microbes during pain crisis hospitalisations (Figure 1) [35, 38, 41]. The dysbiosis in the gut microbiota of individuals with SCD, coupled with the production of inflammatory metabolic products, is believed to impact the pathophysiological aspects of SCD, including the development of chronic pain (Figure 1).

Experiments with SCD mice highlight the role of the gut microbiota in driving chronic pain, as an oral administration of faecal content from these mice-induced pains [14]. Dysbiosis in this population has significant implications, promoting the proliferation of pathogenic bacteria, diminishing beneficial bacteria, and leading to chronic inflammation and immune activation. This inflammatory environment further intensifies the existing inflammation associated with SCD, potentially worsening VOCs by triggering RBC sickling, resulting in pain and a diminished quality of life [34]. In addition, dysbiosis may disrupt the metabolism of common SCD treatments and hinder the absorption of essential nutrients, exacerbating nutritional deficiencies associated with SCD [42].

Addressing dysbiosis in SCD is crucial. Considerations include variations in study design, age, disease severity, geographic and genetic diversity, medication regimen, sampling site, storage, processing, and divergent dysbiosis criteria. Overcoming these limitations is essential for advancing the field and developing targeted interventions.

SCD pathophysiology, marked by RBC sickling and complications such as vaso-occlusion and haemolysis, damages the intestinal epithelium, causing microbial dysbiosis. This, along with factors such as prolonged antibiotic use and nutrient deficiencies, increases gut permeability, promoting pathogenic bacteria and chronic inflammation. Dysregulation exacerbates inflammation, VOCs, and complications in SCD, contributing to pain and organ damage (figure created in BioRender).

### 2.4. Gut Microbiota and SCD Pain

Research on gut microbiota dysbiosis in SCD is evolving, and limitations and variations have been noted in existing studies. While some research suggests that it contributes to specific aspects of SCD, such as chronic pain and bone loss in SCD mice [14, 35], its exact role, however, remains poorly understood. It is unclear whether gut dysbiosis is a contributing factor to SCD pain or a consequence of the underlying disease pathology.

Several studies have attempted to shed light on the mechanisms by which gut microbiota and their metabolites drive chronic pain in SCD.

Evidence supports the role of gut microbes and their metabolites in driving chronic SCD pain by altering vagus nerve activity [14], highlighting the involvement of the gut-brain axis in SCD pain pathophysiology. Dysbiosis in the gut microbiota can lead to a decreased production of short-chain fatty acids (SCFAs), thereby impacting bone health in SCD by reducing IGF-1 [35]. Moreover, gut microbiota dysbiosis in murine SCD is associated with intestinal barrier dysfunction, neutrophilic inflammation, and oxidative stress [40], indicating diverse mechanisms through which the gut microbiota may influence SCD pain.

These findings underscore the intricate interplay between the gut microbiota, their metabolites, and the host in the context of SCD. However, a comprehensive understanding of the specific mechanisms involved in the relationship between gut dysbiosis and SCD pain requires further research. Addressing these knowledge gaps is essential for developing targeted interventions that can improve the management of chronic pain and other aspects of SCD.

## 3. Potential Bacteria Candidates with Therapeutic Implications in SCD

### 3.1. *Akkermansia muciniphila*

*A. muciniphila*, a member of the Verrucomicrobia phylum, is a prevalent bacterium in the human gut. It breaks down and stimulates the production of mucin, a glycoprotein vital for trapping and protecting against pathogens and irritants in the body [43, 44]. Mucin, in turn, enhances gut immunity by producing antimicrobial peptides, increasing mucus thickness, and promoting the gut barrier integrity [45]. Associations between low levels of *A. muciniphila* and various health conditions, including obesity, diabetes, liver steatosis, autoimmune diseases, neurodegenerative disorders, heightened inflammatory responses, and altered efficacy of cancer immunotherapies, have been reported [46–48].

Lower abundance of *A. muciniphila* has been reported in SCD individuals and mouse models [14]. Supplementation of *A. muciniphila* has been found to alleviate SCD-related pain, suggesting a potential role of this bacterium in pain management (Table 1) [14]. The catabolism of bilirubin and biliverdin, breakdown products of haemoglobin by gut bacteria, has been identified as a potential mechanism driving chronic pain in SCD. Oral administration of bilirubin induced widespread vagal nerve-dependent pain in SCD, supporting the idea that manipulating the gut microbiota, particularly by increasing *A. muciniphila* abundance, may be a strategy for pain management in SCD. The study by Sadler et al. [14] is the first to report the effectiveness of *A. muciniphila* in alleviating chronic pain in SCD, offering a nondrug intervention option that could potentially reduce the reliance on opioids and improve patient outcomes.

The precise mechanism by which *A. muciniphila* alleviates chronic pain is not fully understood. However, experimental supplementation with *A. muciniphila* in murine models has shown an increased abundance of mucin-producing goblet cells, thus contributing to the preservation of gut barrier integrity [49]. In vitro studies indicate that *A. muciniphila* enhances enterocyte monolayers' integrity, strengthening the gut barrier [16]. By reducing bacterial translocation and mitigating systemic inflammation and immune activation, *A. muciniphila* could contribute to pain relief in SCD. *A. muciniphila* is involved in the production of SCFAs, including butyrate, known for its anti-inflammatory properties. The outer-membrane protein of *A. muciniphila* activates toll-like receptor 2 (TLR2), regulating inflammation. Moreover, *A. muciniphila* has been observed to induce the transformation of naive CD4+ CD44-Foxp3-T (In) cells into regulatory T (Treg) cell lines, thus playing a role in dampening the excessive immune responses and inflammation in the intestine [15].

In addition, *A. muciniphila* has implications for vascular health, potentially influencing blood flow, oxygen delivery, and pain management. *A. muciniphila* has been shown to facilitate the development of type H vessels, all contributing to the promotion of fracture healing in mice [50]. While seemingly unrelated to chronic pain in SCD, promoting vascular health through *A. muciniphila*, may positively impact blood flow and oxygen delivery. In this population, improved vascular health could alleviate compromised blood flow and oxygenation, potentially reducing the pain associated with VOCs. This underscores the multifaceted potential of *A. muciniphila* in addressing various aspects of SCD, including gut health, inflammation, immune modulation, and now, potentially, vascular health. Overall, *A. muciniphila* shows promise in alleviating chronic pain in SCD by addressing gut barrier dysfunction, reducing inflammation, and modulating immune responses. However, further experimental studies and clinical trials are needed to fully understand the specific mechanisms and their contributions to pain relief.

Recent advancements in microbiome research have identified *A. muciniphila* as a promising candidate for next-generation probiotics [51]. Utilising *A. muciniphila* as a targeted probiotic intervention holds significant potential in ameliorating SCD-related complications and enhancing the quality of life for affected individuals. However, research on *A. muciniphila* strain diversity and its supplementation in various diseases is limited due to challenges in the cultivation and purification of *A. muciniphila* that impede its scalability for therapeutic use. There is also a lack of comprehensive studies on its safety, optimal dosage, and long-term effects in humans. Thus, dietary interventions may enhance *A. muciniphila* abundance and host health. Thus, future research should prioritise investigating the safety, functional diversity, and gut colonisation of *A. muciniphila* strain to improve overall wellness [51].

### 3.2. *Roseburia* spp

*Roseburia* spp. belongs to the *Firmicutes* phylum and the *Lachnospiraceae* family [52]. There are five known *Roseburia* species: *Roseburia intestinalis*, *Roseburia hominis, Roseburia inulinivorans, Roseburia faecis,* and *Roseburia cecicola* [52]. All of these species are primary butyrate-producing bacterial clusters [31] and have been found to have reduced abundance in mouse models and individuals with SCD [13, 38, 40]. Butyrate plays a crucial role in reducing colonic inflammation and oxidative stress, maintaining the gut barrier integrity [53, 54], and inducing the production of HbF [19], which is associated with fewer SCD complications.

Elevated levels of HbF in SCD mitigate the polymerisation of deoxygenated HbS and inhibit RBC sickling and VOCs *in vitro* [19]. It also increases oxygen delivery, improves blood flow, delays the onset of symptoms, and serves as a target for therapeutic interventions to mitigate the disease's severity [55]. Elevated levels of HbF may indirectly alleviate pain in SCD by reducing biliverdin and bilirubin, catabolites of haemoglobin that are elevated in SCD. Bilirubin is directly metabolised by the gut bacteria, and increased circulating levels have been associated with the induction of vagus nerve-dependent pain in both SCD patients and mouse models [14].

Although direct research on *Roseburia spp*. supplementation in SCD is limited, studies on butyrate therapy, a compound primarily produced by *Roseburia spp*., provide insights into potential benefits (Table 1). Butyrate therapy in SCD patients has shown a significant increase in HbF levels, improving overall haemoglobin profiles without adverse effects [19]. In addition, studies using oral sodium 4-phenylbutyrate and intravenous arginine butyrate revealed increased HbF levels without myelotoxicity, indicating a promise as an intervention for SCD patients [56, 57].

The reduced abundance of *Roseburia spp*. in individuals with SCD suggests a potential avenue for improving their quality of life. Increased HbF production stimulated by *Roseburia* spp. may lead to a reduction in SCD-related complications, thereby decreasing the severity and frequency of pain crises, hospitalisations, and blood transfusions [19]. By addressing the underlying causes of complications through gut microbiota modulation, individuals with SCD may experience improved symptoms and require fewer medications and medical interventions, thus enhancing their overall quality of life.

*Roseburia* spp. and its byproduct, butyrate, may mitigate SCD complications through diverse mechanisms. Butyrate suppresses inflammation by inhibiting histone deacetylases (HDACs), thus reducing proinflammatory gene expression [58]. In addition, it influences immune cell function, thereby promoting the development of regulatory T (Treg) cells and potentially balancing the immune response in SCD [20]. Furthermore, butyrate increases the expression of tight junction proteins, maintaining gut barrier integrity and protecting against bacterial translocation, thereby reducing the risk of systemic inflammation [21, 59]. Increased HbF levels induced by butyrate inhibit HbS polymerisation, potentially improving blood flow and decreasing the frequency and severity of VOCs [60].

## 4. Conclusion and Future Perspective

The study's findings suggest that dietary interventions directed toward boosting the presence of *Akkermansia muciniphila* and *Roseburia* spp. carry several important implications for improving the quality of life for this population. Restoring the gut barrier integrity by enhancing mucus thickness and tight junction formation stimulated by *Akkermansia Muciniphila* could be a potential strategy for pain management in SCD. Increasing HbF production, stimulated by butyrate, has the potential to ameliorate SCD complications, reduce the severity and frequency of complications, and decrease the need for hospitalisations, pain episodes, and blood transfusions.

Modulating the gut microbiota offers a sustainable, nondrug approach to managing SCD pain and reducing emergency healthcare reliance. This intervention, beneficial for those seeking alternatives to pharmaceuticals, may decrease opioid usage, thereby improving outcomes and lowering opioid-related risks. Further research on *A. muciniphila* and *Roseburia* spp. is necessary for targeted interventions and understanding their pain-alleviating effects in SCD. Conducting long-term studies will assess *A. muciniphila's* sustained efficacy and any gut microbiota adaptations. Clinical trials should investigate *A. muciniphila* and *Roseburia* spp. supplementation's impact on gut microbiota and SCD clinical outcomes. Personalised treatments based on individual gut microbiota variations in SCD should be explored.

The heterogeneity of SCD poses challenges in devising a one-size-fits-all gut microbiota modulation strategy, given the varied responses, symptoms, and complications. Acknowledging variations in gut microbiota among different ethnicities and geographic locations is crucial, as interventions may not universally apply to diverse groups. The limited number of clinical trials exploring gut microbiota modulation in SCD underscores the necessity for robust evidence to establish safety and efficacy. A critical aspect is understanding the long-term effects of gut microbiota modulation in SCD to assess the durability and potential risks over extended periods.

## Figures and Tables

**Table 1:** Summary of evidence from intervention studies supplementing with *Akkermansia muciniphila* and butyrate.

Author/year	Intervention	Main findings
Sadler et al. 2023	*Akkermansia muciniphila*	This study found that faecal material transplant from SCD mice induced pain in healthy mice, and this pain was linked to bilirubin, a haemoglobin catabolite. Supplementing the gut with *Akkermansia muciniphila* bacteria alleviated chronic pain in SCD mice
Atweh et al.	Arginine butyrate	The study showed that weekly and pulse regimens of arginine butyrate stimulated HbF production in SCD patients. Pulse regimen appeared more effective and better tolerated than the weekly regimen
Dover et al.	Sodium 4-phenylbutyrate	Oral sodium 4-phenylbutyrate increased HbF production in SCD patients and did not appear to cause myelotoxicity as seen with other treatments such as hydroxyurea
Sher and Oliveri et al.	Arginine butyrate	This case report observed that intravenous arginine butyrate led to significant improvements in HbF levels, which may contribute to the complete healing of their leg ulcers in SCD patients

SCD, sickle cell disease; HbF, fetal haemoglobin.

## Additional Points

*Novelty Statement*. What is the new aspect of your work? This work introduces a novel approach by investigating the therapeutic potential of modulating gut microbiota, with a specific emphasis on *Akkermansia muciniphila* and *Roseburia* spp., to alleviate complications and chronic pain linked to SCD. What is the central finding of your work? Supplementing *Akkermansia muciniphila* and *Roseburia* spp. holds promise for addressing SCD complications, notably chronic pain, by targeting gut dysbiosis and promoting gut health through barrier integrity, inflammation reduction, and immune modulation. What is (or could be) the specific clinical relevance of your work? This work suggests a cost-effective approach for managing SCD, offering alternatives to expensive gene therapies. Gut microbiota modulation could improve well-being, reduce reliance on treatments, and offer nonpharmaceutical pain relief for SCD.

## References

[1] da Guarda C. C., Yahouédéhou S. C. M. A., Santiago R. P. (2020). Sickle cell disease: a distinction of two most frequent genotypes (HbSS and HbSC). *Public Library of Science One*.

[2] Quinn C. T. (2016). Minireview: clinical severity in sickle cell disease: the challenges of definition and prognostication. *Experimental Biology and Medicine*.

[3] Ribaldi F., Rolandi E., Vaccaro R., Colombo M., Battista Frisoni G., Guaita A. (2022). The clinical heterogeneity of subjective cognitive decline: a data-driven approach on a population-based sample. *Age and Ageing*.

[4] Gallagher M. E., Chawla A., Brady B. L., Badawy S. M. (2022). Heterogeneity of the long-term economic burden of severe sickle cell disease: a 5-year longitudinal analysis. *Journal of Medical Economics*.

[5] Zaidi A. U., Glaros A. K., Lee S. (2021). A systematic literature review of frequency of vaso-occlusive crises in sickle cell disease. *Orphanet Journal of Rare Diseases*.

[6] Thomson A. M., McHugh T. A., Oron A. P. (2023). Global, regional, and national prevalence and mortality burden of sickle cell disease, 2000–2021: a systematic analysis from the Global Burden of Disease Study 2021. *The Lancet Haematology*.

[7] Galadanci N. A., Umar Abdullahi S., Vance L. D. (2017). Feasibility trial for primary stroke prevention in children with sickle cell anemia in Nigeria (SPIN trial). *American Journal of Hematology*.

[8] Leleu H., Arlet J. B., Habibi A. (2021). Epidemiology and disease burden of sickle cell disease in France: a descriptive study based on a French nationwide claim database. *Public Library of Science One*.

[9] Gbd Sickle Cell Disease Collaborators (2023). Global, regional, and national prevalence and mortality burden of sickle cell disease, 2000-2021: a systematic analysis from the Global Burden of Disease Study 2021. *Lancet Haematol*.

[10] Ware R. E., de Montalembert M., Tshilolo L., Abboud M. R. (2017). Sickle cell disease. *The Lancet*.

[11] Ahmed S. (2023). Challenges with newly approved CRISPR gene technologies. *Molecular Medicine Communications*.

[12] Dutta D., Aujla A., Knoll B. M., Lim S. H. (2020). Intestinal pathophysiological and microbial changes in sickle cell disease: potential targets for therapeutic intervention. *British Journal of Haematology*.

[13] Brim H., Taylor J., Abbas M. (2021). The gut microbiome in sickle cell disease: characterization and potential implications. *Public Library of Science One*.

[14] Sadler K. E., Atkinson S. N., Ehlers V. L. (2023). Gut microbiota and metabolites drive chronic sickle cell disease pain. *bioRxiv*.

[15] Kuczma M. P., Szurek E. A., Cebula A. (2021). Self and microbiota-derived epitopes induce CD4+ T cell anergy and conversion into CD4+Foxp3+ regulatory cells. *Mucosal Immunology*.

[16] Ottman N., Reunanen J., Meijerink M. (2017). Pili-like proteins of Akkermansia muciniphila modulate host immune responses and gut barrier function. *Public Library of Science One*.

[17] Wang J., Xiang R., Wang R. (2020). The variable oligomeric state of Amuc_1100 from Akkermansia muciniphila. *Journal of Structural Biology*.

[18] Wu X., Wu Y., He L., Wu L., Wang X., Liu Z. (2018). Effects of the intestinal microbial metabolite butyrate on the development of colorectal cancer. *Journal of Cancer*.

[19] Atweh G. F., Sutton M., Nassif I. (1999). Sustained induction of fetal hemoglobin by pulse butyrate therapy in sickle cell disease. *Blood*.

[20] Yip W., Hughes M. R., Li Y. (2021). Butyrate shapes immune cell fate and function in allergic asthma. *Frontiers in Immunology*.

[21] Siddiqui M. T., Cresci G. A. M. (2021). The immunomodulatory functions of butyrate. *Journal of Inflammation Research*.

[22] Amabebe E., Robert F. O., Agbalalah T., Orubu E. S. F. (2020). Microbial dysbiosis-induced obesity: role of gut microbiota in homoeostasis of energy metabolism. *British Journal of Nutrition*.

[23] Shama S., Liu W. (2020). Omega-3 fatty acids and gut microbiota: a reciprocal interaction in nonalcoholic fatty liver disease. *Digestive Diseases and Sciences*.

[24] Tomasello G., Mazzola M., Leone A. (2016). Nutrition, oxidative stress and intestinal dysbiosis: influence of diet on gut microbiota in inflammatory bowel diseases. *Biomedical Papers*.

[25] Donaldson G. P., Lee S. M., Mazmanian S. K. (2016). Gut biogeography of the bacterial microbiota. *Nature Reviews Microbiology*.

[26] Stojanov S., Berlec A., Štrukelj B. (2020). The influence of probiotics on the firmicutes/bacteroidetes ratio in the treatment of obesity and inflammatory bowel disease. *Microorganisms*.

[27] Frosali S., Pagliari D., Gambassi G., Landolfi R., Pandolfi F., Cianci R. (2015). How the intricate interaction among toll-like receptors, microbiota, and intestinal immunity can influence gastrointestinal pathology. *Journal of Immunology Research*.

[28] Donati Zeppa S., Agostini D., Ferrini F. (2022). Interventions on gut microbiota for healthy aging. *Cells*.

[29] Gensollen T., Iyer S. S., Kasper D. L., Blumberg R. S. (2016). How colonization by microbiota in early life shapes the immune system. *Science*.

[30] Power S. E., O’Toole P. W., Stanton C., Ross R. P., Fitzgerald G. F. (2014). Intestinal microbiota, diet and health. *British Journal of Nutrition*.

[31] Duncan S. H., Hold G. L., Barcenilla A., Stewart C. S., Flint H. J. (2002). Roseburia intestinalis sp. nov., a novel saccharolytic, butyrate-producing bacterium from human faeces. *International Journal of Systematic and Evolutionary Microbiology*.

[32] Hills R. D., Pontefract B. A., Mishcon H. R., Black C. A., Sutton S. C., Theberge C. R. (2019). Gut microbiome: profound implications for diet and disease. *Nutrients*.

[33] Mohajeri M. H., Brummer R. J. M., Rastall R. A. (2018). The role of the microbiome for human health: from basic science to clinical applications. *European Journal of Nutrition*.

[34] Delgadinho M., Ginete C., Santos B. (2022). Microbial gut evaluation in an angolan paediatric population with sickle cell disease. *Journal of Cellular and Molecular Medicine*.

[35] Xiao L., Zhou Y., Bokoliya S., Lin Q., Hurley M. (2022). Bone loss is ameliorated by fecal microbiota transplantation through SCFA/GPR41/IGF1 pathway in sickle cell disease mice. *Scientific Reports*.

[36] Hrncir T. (2022). Gut microbiota dysbiosis: triggers, consequences, diagnostic and therapeutic options. *Microorganisms*.

[37] Wen L., Duffy A. (2017). Factors influencing the gut microbiota, inflammation, and type 2 diabetes. *Journal of Nutrition*.

[38] Mohandas S., Soma V. L., Tran T. D. B. (2020). Differences in gut microbiome in hospitalized immunocompetent vs. Immunocompromised children, including those with sickle cell disease. *Front Pediatr*.

[39] Zhang J., Ni Y., Qian L. (2021). Decreased abundance of Akkermansia muciniphila leads to the impairment of insulin secretion and glucose homeostasis in lean type 2 diabetes. *Advanced Science*.

[40] Lewis C. V., Sellak H., Sawan M. A. (2023). Intestinal barrier dysfunction in murine sickle cell disease is associated with small intestine neutrophilic inflammation, oxidative stress, and dysbiosis. *Federation of American Societies for Experimental Biology BioAdvances*.

[41] Zhang P. (2022). Influence of foods and nutrition on the gut microbiome and implications for intestinal health. *International Journal of Molecular Sciences*.

[42] Li H., He J., Jia W. (2016). The influence of gut microbiota on drug metabolism and toxicity. *Expert Opinion on Drug Metabolism and Toxicology*.

[43] Belzer C., de Vos W. M. (2012). Microbes inside--from diversity to function: the case of Akkermansia. *The International School of Management Excellence Journal*.

[44] Derrien M., Collado M. C., Ben-Amor K., Salminen S., de Vos W. M. (2008). The Mucin degrader Akkermansia muciniphila is an abundant resident of the human intestinal tract. *Applied and Environmental Microbiology*.

[45] Turner J. R. (2009). Intestinal mucosal barrier function in health and disease. *Nature Reviews Immunology*.

[46] Journey E. K., Ortega-Santos C. P., Bruening M., Whisner C. M. (2020). Changes in weight status and the intestinal microbiota among college freshman, aged 18 years. *Journal of Adolescent Health*.

[47] Xue C., Li G., Gu X. (2023). Health and disease: Akkermansia muciniphila, the shining star of the gut flora. *Research: Ideas for Today’s Investors*.

[48] Zhang Z., Zhang H., Chen T., Shi L., Wang D., Tang D. (2022). Regulatory role of short-chain fatty acids in inflammatory bowel disease. *Cell Communication and Signaling*.

[49] Shin N. R., Lee J. C., Lee H. Y. (2014). An increase in the Akkermansia spp. population induced by metformin treatment improves glucose homeostasis in diet-induced obese mice. *Gut*.

[50] Liu J. H., Yue T., Luo Z. W. (2020). Akkermansia muciniphila promotes type H vessel formation and bone fracture healing by reducing gut permeability and inflammation. *Disease Models and Mechanisms*.

[51] Jian H., Liu Y., Wang X., Dong X., Zou X. (2023). Akkermansia muciniphila as a next-generation probiotic in modulating human metabolic homeostasis and disease progression: a role mediated by gut–liver–brain axes?. *International Journal of Molecular Sciences*.

[52] Nie K., Ma K., Luo W. (2021). Roseburia intestinalis: a beneficial gut organism from the discoveries in genus and species. *Frontiers in Cellular and Infection Microbiology*.

[53] Kim C. H. (2021). Control of lymphocyte functions by gut microbiota-derived short-chain fatty acids. *Cell Molecular Immunology*.

[54] Patterson A. M., Mulder I. E., Travis A. J. (2017). Human gut symbiont Roseburia hominis promotes and regulates innate immunity. *Frontiers in Immunology*.

[55] El Hoss S., Cochet S., Godard A. (2021). Fetal hemoglobin rescues ineffective erythropoiesis in sickle cell disease. *Haematologica*.

[56] Dover G. J., Brusilow S., Charache S. (1994). Induction of fetal hemoglobin production in subjects with sickle cell anemia by oral sodium phenylbutyrate. *Blood*.

[57] Sher G. D., Olivieri N. F. (1994). Rapid healing of chronic leg ulcers during arginine butyrate therapy in patients with sickle cell disease and thalassemia [letter]. *Blood*.

[58] Davie J. R. (2003). Inhibition of histone deacetylase activity by butyrate. *The Journal of Nutrition*.

[59] Yan H., Ajuwon K. M. (2017). Butyrate modifies intestinal barrier function in IPEC-J2 cells through a selective upregulation of tight junction proteins and activation of the Akt signaling pathway. *Public Library of Science One*.

[60] Manwani D., Frenette P. S. (2013). Vaso-occlusion in sickle cell disease: pathophysiology and novel targeted therapies. *Blood*.

